# Comparative Assessment of Public Opinion on the Landscape Quality of Two Biosphere Reserves in Europe

**DOI:** 10.1007/s00267-014-0316-9

**Published:** 2014-07-04

**Authors:** Barbara Sowińska-Świerkosz, Tadeusz J. Chmielewski

**Affiliations:** Department of Landscape Ecology and Nature Conservation, University of Life Sciences in Lublin, Dobrzańskiego 37, 20-262 Lublin, Poland

**Keywords:** Comparative regional studies, European Landscape Convention, Landscape evaluation, Landscape quality

## Abstract

The European Landscape Convention (2000) obligates European Union countries to identify and implement landscape quality objectives (LQOs) understood as the specification of public expectations and preferences concerning the landscape of a given area, expressed by competent public authorities. The convention emphasizes the important role of local community representatives in this field. In Poland, the implementation of the LQO concept was first undertaken in two regions with radically different landscape characteristics: (1) the West Polesie Biosphere Reserve and (2) the selected protected areas of the Roztocze–Solska Forest, nominated to the rank of a biosphere reserve. The first stage of the presented study was the recognition of public opinion on the quality of key features of landscape, based on a questionnaire (*n* = 470). The primary objective of the study was to provide an answer to the following questions: (1) Whether similar social expectations regarding landscape quality exist in spite of radically different landscape characteristics of the regions investigated (landscape quality is understood as spatial arrangement, scenic beauty, and lack of environmental pollution); (2) which landscape features are considered to be most preservation worthy by the representatives of both local communities; and (3) What processes or development impacts pose the greatest threat to the landscape quality of both regions according to the public opinion? The conducted comparative assessment revealed that it is possible to define a set of features fundamental to the quality of both areas and that representatives of local communities pointed out the same threats to the natural and cultural values of both regions investigated.

## Introduction

The European Landscape Convention (2000) obligates its signatories to identify characteristic landscape features occurring within the territories of individual member states to assess their values, analyze the forces and pressures transforming them, and define landscape quality objectives (LQOs). The convention defines these objectives as “the formulation by the competent public authorities of the aspirations of the public with regard to the landscape features of their surroundings” (art. 1, paragraph c). They should be defined for specific and characteristic landscapes of individual regions. The primary goal of LQOs was to secure high quality of landscapes on the European continent by the authorities of each country in collaboration with local communities (Chmielewski and Sowińska 2010). This requires determining which qualities are most worthy of concern (Arler 2000).

Landscape quality may be considered from either an objective or a subjective perspective (Arler 2000; Lothian 1999). According to an objective approach, landscape quality should be measurable and comparable and can be indirectly classified and marked on a map of land relief, soil cover, or vegetation. Research of this type is generally based on itemizing the so-called substitute indices of quality, reflecting the quality of each landscape component in quantitative terms (Hendriks et al. 1997; Kuiper 1998). Ecological indicators are commonly applied, providing information on the resources, degree of cleanliness, and tolerance to anthropopressure of the abiotic and biotic components of the environment (Schiller et al. 2001). Landscape metrics are also calculated in order to define the quality of landscape. These indices enable researchers to quantitatively reflect features regarded as crucial to the quality of landscape, such as spatial configuration, density, richness, and diversity of landscape patches (Dale and Beyeler 2001; Schiller et al. 2001).

On the other hand, landscape quality has a subjective dimension, depending on an individual opinion of each observer, his psychological profile and environmental experiences. According to this approach, the assessment of visual components of landscape is carried out by representatives of various socio-professional groups of people (Bulut and Yilmaz 2008; Cañas et al. 2009; Tveit 2009). The following methods are commonly applied in this approach: public opinion poll, the photograph evaluation, face-to-face or over-the-phone interview, indoor group discussion, discussion in the field, and internet-based systems (among others: Sevenant and Antrop 2009; Barroso et al. 2012; Roth 2006; Tveit 2009).

The subjective approach permits us to determine the way landscape was perceived in the past, its perception in the present, the components most essential in the perception process, as well as trends considered to be most significant to landscape quality. It also permits the prediction of the future landscape, indirectly developed by present generations (Lothian 1999). The analysis of relationships between human behavior and the natural or man-made environment constitutes an important aspect of the subjective approach as well. Considering the definition of LQOs, as well as the concept of landscape as defined in the European Landscape Convention, i.e., “an area, as perceived by people, whose character is the result of the action and interaction of natural and/or human factors (art. 1, paragraph a),” the subjective approach appears appropriate to define these objectives. Landscape as a public good should reflect the needs of an extensive group of people. Moreover, legal actions undertaken in this field, according to sociological studies, are likely to be successful because they have public support and reflect the needs of inhabitants concerning the directions of spatial development (Luginbüh 2006). Furthermore, according to the European legislative requirements, all actions aimed at defining, applying, and monitoring landscape policies should be preceded and accompanied by procedures involving participation by members of the public and other relevant stakeholders. The goal is to enable them to play an active role in formulating and monitoring the quality of landscape components (Jones 2007; Recommendation CM/Rec 2008).

The Council of Europe Guidelines (Recommendation CM/Rec 2008) proposes a wide range of participatory methods, stressing the exchange of ideas between local people affected by spatial planning on one hand, and scientists and experts possessing technical knowledge on the other. Sociological studies were carried out in different countries by representatives of various fields of science. A survey questionnaire was generally used in cases related to the LQO concept (Chmielewski and Sowińska 2008; Jones 2007; Nague and Sala 2006; Olmo et al. 2006; Sevenant and Antrop 2010; Sowińska and Chmielewski 2006). This tool permits the determination of public opinion on landscape preference, as well as environmental attitude and behavior.

Loupa (2010) recommends another feasible approach to LQO identification through sociological research. She suggests the use of exploratory landscape scenarios, ordering one’s perceptions of alternative futures. For this purpose, various visualization techniques were developed, such as drawings, walk-through or fly-through animations, digital simulation using GIS and 3D tools, and photorealistic representations. In the case study of the Mėrtola region of Southeast Portugal, these techniques were used to visualize the plausible futures of landscapes in 2035 (Loupa 2010). A similar approach was used in a Swiss project called ‘Paysage 2020’ (Landscape 2020) (Stalder 2006). In England, a specific virtual landscape model of the Alport Valley was used, consisting of a digital terrain model, an orthophotomap, and objects such as trees, dry stonewalls, buildings, paths, and the sky as a backdrop (Lange and Hehl-Lange 2010). The application of visual simulation revealed that these are practical tools to predict plausible futures rather than optimal ones, which can help identify alternative drivers of change.

Regardless of various research tools, the participatory approach to the identification of LQOs seems to be the optimal way to apply them in practice as an effective tool for the conservation and design of different types of landscapes. Since the elaboration of LQOs should be done in close cooperation with the inhabitants of particular areas, the most suitable spatial scale for work of this type appears to be the sub-regional scale, comprising mezoregions and groups of physiographic mezoregions, individual protected areas (such as national parks, landscape parks, or biosphere reserves), compact systems of protected areas, or cultural regions.

Research on LQOs undertaken so far in different countries was conducted in relation to one natural region or administrative area (Naguė and Sala 2006; Chmielewski and Sowińska 2010; Ramos 2010). A comparative analysis LQOs identified for different regions has not been done so far, and there are only a few comparative studies that reflect public preferences for different types of landscapes (for example, Sevenant and Antrop 2009). Such analyzes demonstrate to what extent the public opinion depends on the landscape type and whether it is possible to distinguish a set of common features considered crucial for landscape quality. Distinction of such a set of features could facilitate the process of land management by making it possible to shift the LQO studies from the regional to national level by using homogeneous criteria for the entire territory of a given country. However, apart from the great importance of landscape quality issue, little research on public opinion on this subject has been conducted in Poland.

The goal of the present article was to fill this gap by conducting a comparative analysis of the public opinion on landscape quality of two regions with radically different character of landscape and to provide answers to the following questions:

1. Whether similar social expectations regarding landscape quality exist in spite of radically different character of landscape of the two regions investigated;
2. if so, which landscape features are considered to be most preservation worthy by the representatives of both the local community and tourists;
3. which activities and processes or development impacts pose the greatest threat to the landscape quality of both regions according to the public opinion.

An important methodological requirement based on the objectives of the study was to compare public opinion on landscape quality with respect to two contrasting study areas, both of very high (world class) natural and cultural values. Thus, two biosphere reserves have been selected: the West Polesie Biosphere Reserve and the Roztocze–Solska Forest protected areas cluster, nominated to the status of a biosphere reserve. The choice was also justified by the statutory documents of the UNESCO MAB (Man and Biosphere) Program, introducing the World Network of Biosphere Reserves (BR). This document suggests that a public participation approach should be applied in the management of these protected areas.

### Study Area

**Fig. 1:**
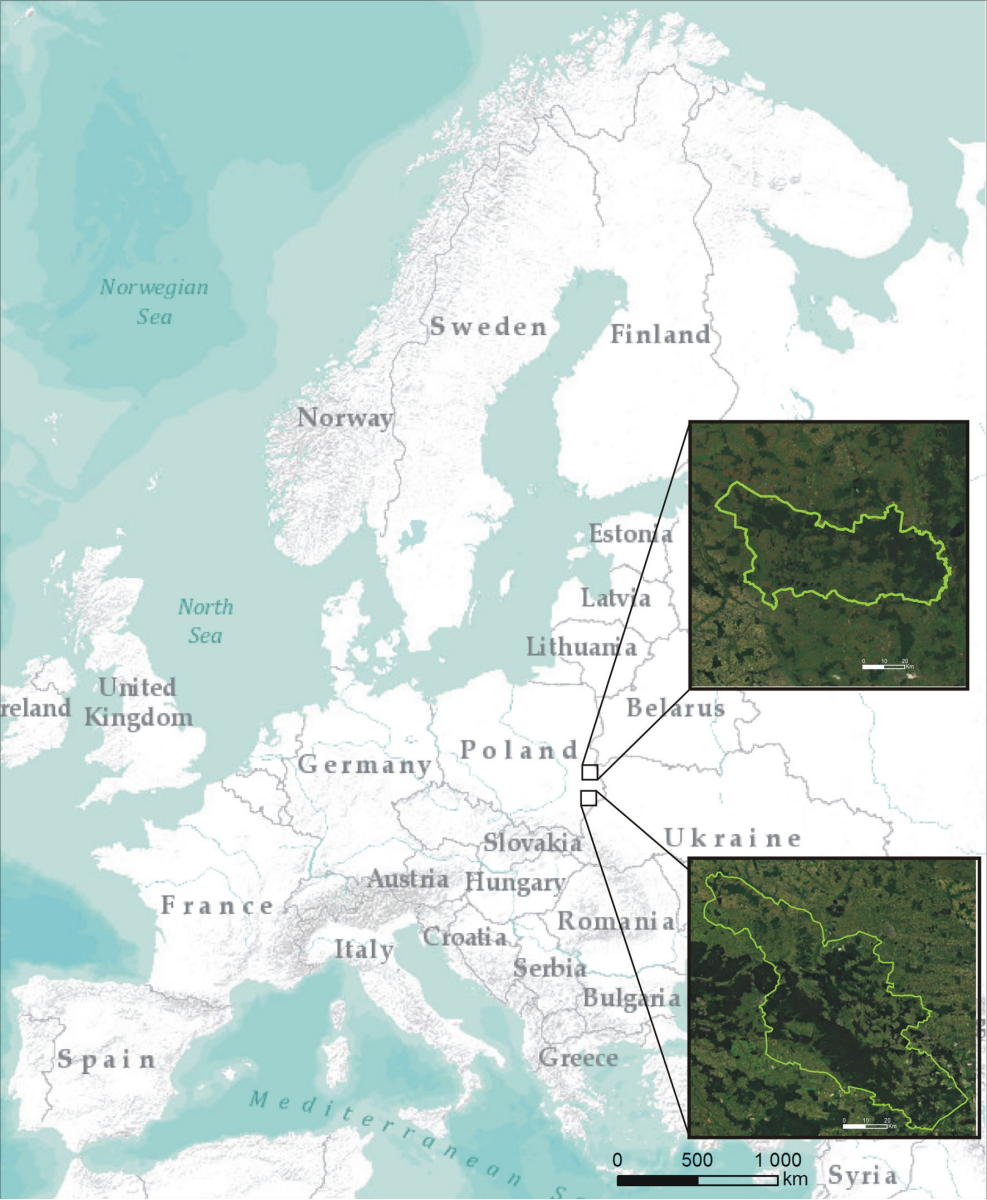
Location of both study areas on the background of Europe and their borders on the background of orthophotomap

**Fig. 2:**
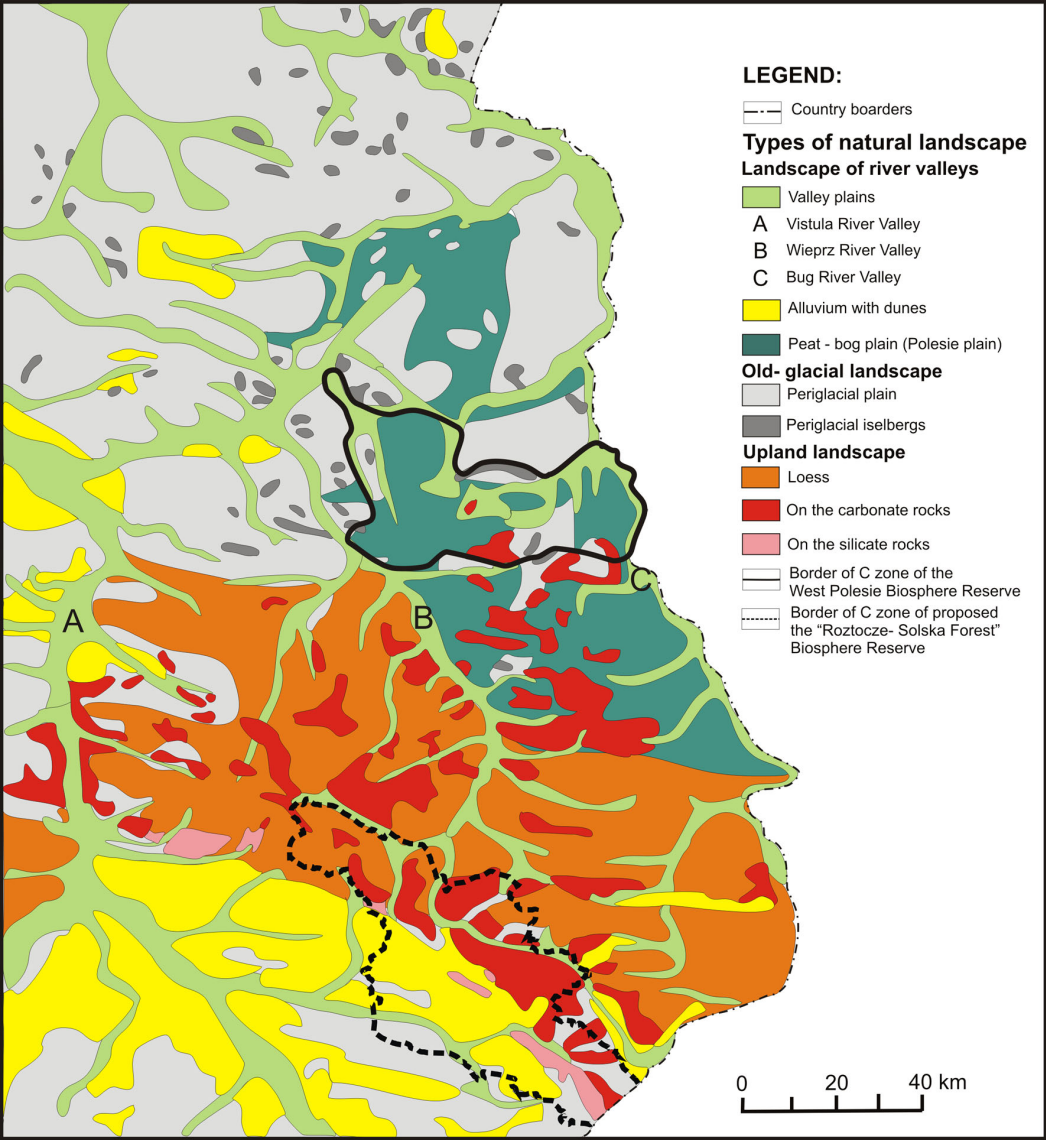
Location of the biosphere reserves on the background of types of natural landscape [typology elaborated by Richling and Ostaszewska (2005)]

**Fig. 3:**
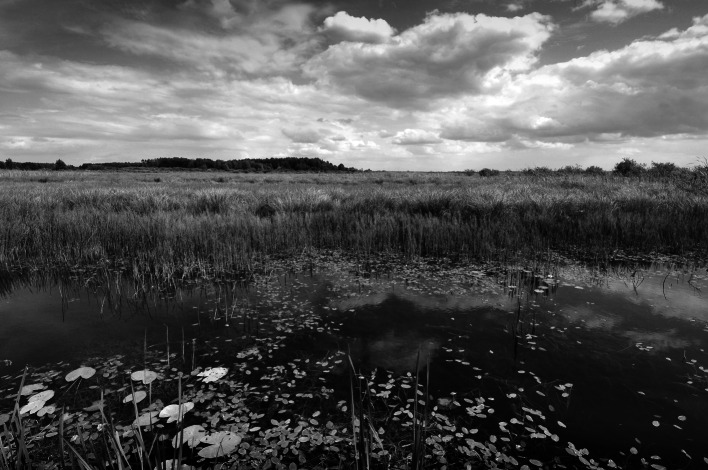
Bubnów peatbog in the West Polesie Biosphere Reserve

Both study areas are located in central eastern Poland near the EU border with Belarus and Ukraine (Fig. 1). The West Polesie BR was established in 2002 on an area of approximately 1,400 km^2^. The reserve comprises old glacial landscape characterized by lowland, and flat, wetland areas (Fig. 2). The characteristic elements of the Polesie region are 61 lakes usually surrounded by peatbogs (Fig. 3) and forests. The land use structure of the West Polesie BR includes: 59.5 % forests (where 30 % are located on wet and marshy habitats); 25.3 % fields and buildings; 7.8 % meadows (where wet grasslands occupy over 6 %); 2.8 % water terrains; 2.4 % peatbogs; and 2.2 % shrubs and fallow land. The Polesie flora is characterized by a large number of northern plant species (150 species) and simultaneous presence of many plants from the Atlantic zone (25 species), and east continental zone (43 species) (Chmielewski 2005). The animal life is also abundant here. According to the research, there are over 300 species of aquatic non-vertebrates, 35 species of Ichthyofauna, and at least 150 breeding species of avifauna. Among reptiles, the mud turtle (*Emys orbicularis*) is an example and among mammals the otter, wolf, and elk. In the 50s and 60s of the twentieth century, the Polesie region was subject to an excessive drainage process which resulted in lowering the water level by 1 m and the disappearance of about 73 % of wetlands. Since the 70s, lakes of the region have been subjected to invasive tourism pressure, especially the expansion of tourist infrastructure. Over the last 40 years, the developed area has increased by 570 ha (55.4 %) (Chmielewski and Chmielewski 2010). Despite those pressures, many areas have preserved their high natural values. The West Polesie BR includes the Polesie National Park, four landscape parks, nine Natura 2,000 sites, and 10 natural reserves (Chmielewski 2005).

**Fig. 4:**
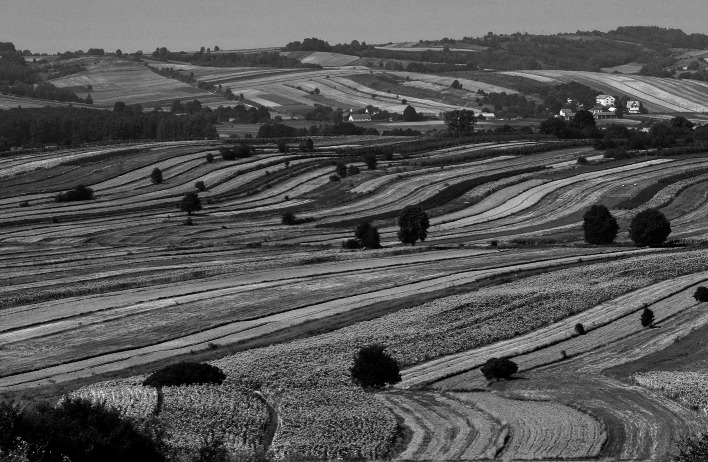
Typical rural landscape of the West Roztocze region

The future Roztocze–Solska Forest BR covers an area of approx. 2,400 km^2^. It is located within two large European structural units: the east-European platform consolidated in the pre-cambrian era and the orogenic Paleozoic structures of western Europe. The region is also located within the European water division separating the Vistula river system with catchment area in the Baltic Sea from the Dniestr river system with catchment area in the Black Sea (Sowińska and Chmielewski 2011). It is predominantly covered with complexes of multi-species forest and a multi-stripe and multi-color field mosaic, with lines of numerous balks overgrown with a variety of weeds and numerous clusters of trees and shrubs (Fig. 3). The future biosphere reserve has a very diverse landscape. It is distinguished by the occurrence of loess uplands with a dense network of ravines, sloping carbonate hills, accumulation plains with dunes, and small river valleys (Fig. 4). The land use structure the projected BR includes: 55 % forests (among which 63 % are coniferous, 7 % deciduous, and 30 % mixed), 28 % fields, 8 % developed areas, 5 % grasslands, 3 % waters terrains, and 1 % peatbogs. Due to its ecotone location between the Roztocze and Biłgoraj plain, the BR has a unique abundance of flora. It provides natural habitat for over 900 species of vascular plants, present mainly in the forest and meadow-bog communities, including nearly 70 rare taxa and about 200 synanthropic species. Moss flora is represented here by nearly 200 species, mushrooms by over 1,000 species, and biota of lichens by about 300 species. With the exception of agrocenosis, more than 120 plant communities have been identified here. Equally rich and diverse are the fauna world of the planned Reserve. According to research, there are about 3,500 species of invertebrates and 372 of vertebrates (Chmielewski 2004). The land use structure of the Roztocze–Solska Forest region is very dynamic. They include the expansion of buildings over open fields, and the fields overgrowing with vegetation in some parts of the region. The system of protected areas of the future BR is composed of the Roztocze National Park, four landscape parks, 15 nature reserves, 19 Natura sites, one landscape protected area, and more than 30 ecological lands (Sowińska and Chmielewski 2011).

### Methods

In Poland, studies of the development of methodological framework for LQOs identification started in 2006. It was assumed that LQOs should include three main components: 1. characteristic landscape features worthy of preservation (landscape canon); 2. other expected features concerning landscape quality (objectives to be attained); and 3. methods for the achievement and conservation of the desired status of landscape (guidelines and instruments) (Chmielewski and Sowińska 2010; Sowińska and Chmielewski 2011).

According to the approach adopted and the requirements of the European Landscape Convention, the first stage of LQOs identification should be the recognition of the public opinion and expectations concerning the values and threats to the natural and cultural heritage, as well as the landscape quality expected or desired by the inhabitants and tourists. Research in this field was performed simultaneously in two areas: (1) the West Polesie BR and (2) the Roztocze–Solska Forest BR, and it concerned two issues: (a) public opinion on landscape quality and (b) public opinion on the threats to landscape quality.

The research was divided into three stages: (1) sociological research; (2) systematization of landscape features and identification of the threats to landscape quality; and (3) comparative analysis of the public opinion on the landscape quality and its threats.

### Sociological Research

The main objective of the sociological research was to determine the public opinion on the most characteristic landscape features that should be conserved and considered when formulating the LQOs for the two BRs investigated. The method of an opinion poll was applied. The content of the questionnaires was the same for both BRs. The poll covered eight social and occupation groups, playing key roles in the protection and development of landscape in each BR. The groups included: (1) farmers; (2) expert–scientists; (3) employees of the national park, landscape parks, and Public Forests; (4) representatives of local governments; (5) tourists and owners of summer houses; (6) members of pro-ecological and art organizations; (7) teachers working in the researched regions; and (8) college students from those regions. The expert–scientist group included university staff, i.e., natural sciences specialists who had conducted research in the areas under study and thus possess unique knowledge about natural and cultural values of those regions. The local governments representative group contained employees of municipalities who had been elected in local free elections. Their powers included, in particular, the preparation of spatial development plans. The administration of nature conservation employees and public forest staff is state professionals who deal directly with the management of protected areas or forest complexes. We also decided to select a group of people belonging to pro-ecological and art organizations, since they may perceive landscape in a different way from other groups. They are particularly sensitive to landscape beauty and its picturesque quality. They seek harmony between landscape components and are exceptionally perceptive of disharmonious elements. The selection of teachers as a separate occupation group was due to their significant influence on the environmental attitudes of the younger generation. Furthermore, in the rural areas of Poland, a teacher being a person with a college degree, belongs to “the social elite.” The last group included college students majoring in nature conservation, landscape architecture, and environmental engineering, who come from the two researched BRs. They will in the future determine the character of spatial development and wildlife conservation in these regions.

The questionnaires were directly distributed to random persons representing the first four groups during public events, such as scientific conferences, national park board meetings, board meetings of the assembly of landscape parks, assemblies of landscape photography association, meetings of pro-ecological organizations, school councils, and classes at the university. In the case of farmers and tourists, the questionnaire was distributed to random persons casually met in the fields in different parts of each region. In all the cases, the work was generally carried out by the authors of the manuscript who also verbally explained the reason and the goal of undertaking the research.

All the questionnaire participants were asked two kinds of questions: (1) Which landscape characteristics of the West Polesie/Roztocze–Solska Forest Biosphere Reserve do you consider to be worthy of conservation? and (2) What are the major threats to the characteristic landscape features of the West Polesie/Roztocze–Solska Forest Biosphere Reserve? In both cases, the participants were asked to use a 5-point scale to rate the listed features according to their landscape preferences and their knowledge about the studied terrain, with five points ascribed to the feature considered most important for the quality of landscape in a given area and one point to the feature regarded as the least important. A letter of introduction was attached to the questionnaire. It referred to the ELC as a justification of research on landscape quality preference.

The opinions of 230 persons were collected from the West Polesie region (30 people for each social group, except the pro-ecological and art organizations group, represented by only 20 persons) and 240 persons from the Roztocze–Solska Forest region (30 people for each social group).

One-way analysis of variance and independent means *t* tests were used to compare the scores given by different groups of respondents. The comparison was applied only to those characteristics and threats which were listed and rated by representatives of all eight groups, a total of 12 features and four threats from each reserve.

### Systematization of Landscape Features and Its Threats

In the second stage of the study, landscape features and landscape threats listed by the participants were assigned to the appropriate landscape components. A list of elements determining the landscape identity and fundamental to its quality according to public opinion was established. Subsequently, landscape features were classified into major categories of components. The character of categories resulted from a generally accepted definition of landscape, which depicts it as a vast, complex spatial system comprising three mutually connected hierarchical subsystems: (1) abiotic, (2) biotic, and (3) cultural. The visual effect of the coexistence of these three constituents is (4) landscape physiognomy typical of a given area and directly perceived by people (Chmielewski 2012). Thus, the features crucial to the quality of landscape amounted to four categories.

The main goal of this systematization procedure was to present the landscape features and threats listed by the respondents in an orderly and hierarchical way. Such classification was necessary to carry out the comparative analysis of the public opinion on landscape quality in two regions with radically different landscape characteristics, following in the next stage of the study.

### Comparative Assessment of the Public Opinion on the Quality of Landscape and Its Threats

Initially, the percentage of the total number of points attributed by respondents from each BR to a particular landscape feature was calculated. The next step was defining the so-called Feature Importance Index (FII), which describes the public rank of each landscape feature to be preserved. The lowest numerical value was ascribed to features not mentioned by the respondents or given a low number of points, and the highest value to the features given the most points. The same method was adopted for the purpose of developing a ranking of threats to the quality of landscape in both BRs. Furthermore, the Threat Rank Index (ThRI) illustrating the public evaluation of dangers threatening to each landscape component was calculated. The lowest numerical value was attributed to the component considered to be least threatened, and the highest values to the element regarded as most endangered.

To comparatively assess the quality of landscape features, the Total Feature Importance Index (TFII) was also calculated, as the sum of the FIIs calculated for both reserves with reference to each landscape feature evaluated. This purpose of this procedure was to determine which landscape features are considered most important by both local communities. Providing an answer to this question constitutes one of the objectives set by the authors at the beginning of the present study.

## Results

### Sociological Research

In the case of the West Polesie BR, representatives of the local community described almost 40 landscape features considered as crucial for landscape quality of this region and requiring conservation (Table 1, Column 3). The highest number of points was ascribed to: (1) the abundance and natural state of lakes; (2) high biological diversity (including the mosaic of various types of ecosystems); (3) inaccessible, virgin lakes; and (4) regional wooden rural architecture with traditional gardens.

**Table 1:** Comparative assessment of social opinion on the landscape quality of the West Polesie Biosphere Reserve and the future Roztocze–Solska Forest Biosphere Reserve

1. Category of components	2. Components of landscape	West Polesie			Roztocze–Solska Forest			9. Total Feature Importance Index
		3. Social opinion on the most characteristic landscape features which should be conserved	4. % of the total number of points	5. Feature Importance Index	6. Social opinion on the most characteristic landscape features which should be conserved	7. % of the total number of points	8. Feature Importance Index	
Abiotic	1. Local climate	No comment	–	1	Pleasant microclimate, air rich in essential oils	0.12	4	5
	2. Natural relief	Plain, monotonous, lowland areas	7.30	12	Characteristic, undulating, upland landscape	7.41	19	31
	3. Geomorphological and geological forms	Clearly identified geomorphological forms of glacial origin	0.02	3	Outliers, fossils, and glacial erratic	0.54	12	15
					Unique features of geological scarps	0.05		
						Σ 0.59		
	4. Springs and rivers	Natural state of river valleys	0.09	6	Plentiful of springs and clean rivers	7.42	23	29
					Natural state of river valleys	0.91		
						Σ 8.33		
	5. Lakes and ponds	Abundance and natural state of lakes	9.12	20	Small water bodies located in fields	0.18	9	29
		Inaccessible virgin lakes	8.90		Presence of fish ponds	0.16		
		Lakes as natural fisheries	0.01			Σ 0.34		
			Σ 18.03					
	6. River gorges	No comment	–	1	Unique river gorges constituting a typical element of the West Roztocze landscape	0.66	13	14
	7. Dunes	Sparse, poorly developed dunes	0.01	2	Natural sand dunes	0.06	2	4
	8. Loess ravines	No comment	–	1	Dense network of loess ravines and gullies	0.81	16	17
Biotic	9. Wetlands and peatbogs	Natural, not drained wetlands and peatbogs	8.56	16	Small peatbogs and swamps located in forests	0.17	6	22
	10. Meadows	Vast open areas of meadows	5.95	11	Meadows with various species of flowers	0.09	3	14
	11. Forests	Vast complexes of diverse natural forests	8.19	15	Vast complexes of diverse natural forests	8.18	22	37
	12. Species of fauna and flora	Protected species of fauna and flora	0.06	7	Mainstays of rare species of fauna and flora	0.24	8	15
		Rare species of migrant birds	0.04		Xerothermic species	0.09		
			Σ 0.10			Σ 0.33		
	13. Biological diversity	Very high biodiversity, unique richness of fauna and flora habitats	9.08	18	High diversity of flora and fauna species	0.71	15	33
		Diversity of landscape forms	0.64		Richness of butterflies on meadows	0.09		
			Σ 9.72			Σ 0.80		
Cultural	14. Rural landscape	Traditional rural landscape	0.08	5	Multi-stripe field mosaic with lines of numerous balks overgrown with weeds and numerous clusters of trees and shrubs	8.84	26	31
					Historical fields structure of West Roztocze	7.83		
					Agricultural landscape with no buildings or technical infrastructure	0.76		
					Balks and trees in the fields	0.52		
					Afforestation along roads and around farms	0.34		
					Natural surface of country roads	0.04		
					The landscape of harvest time	0.01		
						Σ 18.34		
	15. Rural houses and house gardens	Regional wooden rural architecture with traditional gardensSmall area, rural, and provincial colonizationWild fruit orchards	8.63	17	Regional wooden rural architecture	6.90	24	41
			0.02		Small area rural and provincial colonization	6.05		
			0.01		Traditional orchards and gardens	0.32		
			Σ 8.66		Traditional fences	0.03		
					Traditional spatial village structure	0.03		
						Σ 13.33		
	16. Churches and chapels	Numerous churches constituting living evidence of the ages-long coexistence of three cultures and religions: Catholic. Orthodox, and Judaist	8.12	14	Religious sanctuaries, monuments of religious architecture, and collection of small roadside and riverside chapels	7.74	21	35
					Orthodox churches and synagogues	0.24		
					Trees around sacral building	0.05		
					Highly valuable church complexes	0.02		
						Σ 8.05		
	17. Monuments of architecture and historical sites	Historical sites, particularly those related to the January uprising and the World War II	7.57	19	Local museums and outdoor museums	0.48	17	36
		Remains of historical park complexes and granges	7.49		Historical cemeteries and gravestones	0.33		
		Monuments of architecture	0.26		Historical sites related to wartimes	0.16		
		Archaeological sites	0.09		Historical industrial buildings	0.09		
			Σ 15.41		Archaeological sites	0.01		
						Σ 1.07		
	18. Local tradition	Traditional customs and occupations	0.08	5	Traditional customs, occupations, folk groups, and dishes	0.22	7	12
	19. Other typical elements of cultural heritage	Water cranes and windmills	0.02	4	Historical urban-landscape structures of the former Zamoyski Estate Quarries	7.86	20	24
		Remains of non-existent villages	0.01		Avenues of trees and manors’ parks	0.02		
		Quarries	0.01			0.12		
			Σ 0.04			Σ 8.00		
Scenic beauty	20. Diversity of land use patches	Rich mosaic of small patches of water, peatbog, meadow, and field ecosystems	7.81	13	Diverse mosaic of small patches of fields and forests	8.21	25	38
					Diverse mosaic of forest, peatbog, meadow, and steppe ecosystems	6.98		
					Diversity of landscape forms	0.07		
						Σ 15.26		
	21. View openings	No comment	–	1	Vast open spaces of fields	5.91	18	19
					Numerous view points	0.10		
						Σ 6.01		
	22. Picturesque landscape	Picturesque location of lakes	0.27	8	Picturesque flora	0.04	1	9
		Picturesque flora	0.05					
			Σ 0.32					
	23. Harmony of landscape forms	Harmonious coexistence of buildings and technical infrastructure in the rural landscape	0.02	3	Harmonious coexistence of buildings and technical infrastructure in the rural landscape	0.08	5	8
		Vast areas of landscape without anthropogenic elements	0.01		Landscape without anthropogenic elements	0.08		
			Σ 0.03			Σ 0.16		
Other characteristic elements	24. Agriculture	Traditional species of crop plants and farming animals	0.01	2	Traditional species of crop plants and farming animals	0.56	14	16
					Ecological farming	0.17		
					Diversity of cultivations	0.04		
						Σ 0.77		
	25. Protected areas	Different types of protected areas of fauna and flora species	0.84	10	Vast protected areas	0.32	11	21
		Numerous monuments of nature	.07		Numerous monuments of nature	0.15		
			Σ 0.91		Ecological corridors	0.02		
						Σ 0.49		
	26. Tourist infrastructure	Parking places and access roads to lakes	0.22	9	Tourist cycle and walking trails	0.27	10	19
		Tourist trails	0.20		Ecotourism	0.07		
		Tourist resorts	0.14		Health resorts	0.03		
			Σ 0.56			Σ 0.37		

Differences of opinion between groups of respondents with regard to most characteristic landscape features of Polesie region were statistically significant (Table 2, part A). Only opinions relating to the first feature category: the abundance and natural state of lakes, as well as natural, not drained wetlands and peatbogs did not show much variation. The biggest difference in means occurred with reference to vast complexes of diverse natural forests (max difference = 2.83) and plain, monotonous, lowland areas (max difference = 2.50). In both cases, those features received the lowest number of points in the farmers’ opinion and the highest in the tourists’ opinion. It may be due to the fact that familiarity with landscape types and elements strongly influences landscape preferences (Kaplan and Kaplan 1989). The biggest discrepancy in opinions was observed between farmers and the majority of other groups, such as expert–scientist, representatives of local governments, tourists, and students.

**Table 2:** Attitudes of West Polesie Biosphere Reserve respondents toward main landscape features and its threats

Factor	1	2	3	4	5	6	7		8	*F* (7,222)	Significant difference between means			Significance *P*<
											*P* < 0.05	*P* < 0.01	*P* < 0.001	
A. Main characteristic landscape features														
Abiotic features														
Plain, monotonous, lowland areas	2.40	3.23	3.07	3.53	4.23	4.20	3.00		1.73	12.09	1–2 4–5	1–4 3–6 1–8 6–7 2–5	1–5 3–8 6–8 1–6 4–8 7–8 2–8 5–7 3–5 5–8	.000
Abundance and natural state of lakes	3.53	3.87	3.70	3.73	4.23	3.90	4.20		4.13	1.27	–	–	–	n.s.
Inaccessible virgin lakes	2.47	3.73	3.17	3.47	4.77	4.55	4.20		4.43	17.49	1–3 2–8 4–7 5–6	1–4 2–6 4–8 5–7	1–2 1–8 3–7 1–5 2–5 3–8 1–6 3–5 4–5 1–7 3–6 4–6	.000
Biotic features														
Natural, not drained wetlands and peatbogs	3.13	4.10	3.40	3.87	3.87	3.70	3.53		3.60	1.57	–	–	–	n.s.
Vast open areas of meadows	1.80	2.53	3.33	3.47	2.53	2.95	2.40		2.33	5.12	1–2 3–5 1–5 3–8 2–3 7–8	2–4 4–8 2–7 5–7 4–5	1–3 3–7 1–4 4–7 1–6 6–7	.000
Vast complexes of diverse natural forests	1.60	3.48	3.27	3.60	4.43	2.85	4.33		4.43	23.07	4–6 4–7	2–7 3–7 4–5	1–2 1–7 5–6 1–3 1–8 6–7 1–4 2–5 6–8 1–5 2–8 1–6 3–8	.000
Very high biodiversity, unique richness of fauna and flora habitats	2.97	4.70	3.63	3.07	4.57	2.85	4.13		4.70	14.83	1–3 5–7 7–8	4–7 6–7	1–2 2–6 4–5 1–5 2–7 4–8 2–3 3–5 5–6 2–4 3–8	.000
Cultural														
Regional wooden rural architecture with gardens	3.00	4.07	3.40	3.93	4.00	4.90	3.40		3.33	5.82	1–4 2–7 2–3 2–8	1–2 1–5	1–6 4–6 6–8 2–6 5–6 3–6 6–7	.000
Churches constituting living evidence of the ages-long coexistence of three cultures	2.80	2.80	3.17	3.03	3.87	3.90	4.47		4.00	7.54	3–5 4–6 3–6 4–8 4–5 6–7	1–6 2–6 2–5 2–8	1–5 2–7 4–7 1–7 3–7 1–8 3–8	.000
Historical sites	2.60	3.23	3.27	3.67	3.53	2.25	3.00		4.10	4.34	1–5 2–8 2–6 3–8	1–4 7–8 5–6	1–8 6–8 4–6	.000
Remains of historical park complexes and granges	2.20	3.37	3.77	3.13	4.23	2.65	2.80		3.37	7.53	1–7 6–8	1–4 3–7 2–5 4–5 3–6 5–8	1–2 1–8 1–3 5–6 1–5 5–7	.000
Scenic beauty														
Rich mosaic of small patches of water, peatbog, meadow and field ecosystems	2.53	4.30	3.03	3.27	4.23	2.95	3.13		3.23	7.16	1–4 1–8	–	1–2 2–6 5–6 1–5 2–7 5–7 2–3 2–8 2–4 4–5	.000
B. Main threats to characteristic landscape features														
Disappearance of wetlands, drying of peatbogs and bogs, as well as regulation of river beds	3.00	4.43	3.77	4.07	3.97	2.30	4.47	4.13		5.82	1–3 2–3 2–4 3–7	1–4 1–5 1–8	1–2 4–6 1–7 5–6 2–6 6–7 3–6 6–8	.000
Location of tourist housing on lakesides	2.67	3.77	3.27	3.27	4.30	3.90	3.53	4.27		6.12	1–3 3–6 1–4 3–6	1–2 5–7 4–5 7–8 4–8	1–5 1–8 1–6 3–5 1–7 3–8	.000
Disappearance of open-space peatbogs and meadows, taken over by forests and construction	2.33	3.17	3.27	3.07	3.93	2.00	3.53	3.30		5.92	1–2 2–5 1–4 4–5	1–3 4–6 1–8 6–8 2–6	1–5 5–6 1–7 6–7 3–6	.000
The sprawl of buildings and summer cottages over the open space of fields and meadows	2.47	3.53	2.90	3.67	3.93	2.85	2.67	4.00		6.39	2–7 4–6 3–4	1–2 4–7 3–5 5–6 3–8 6–8	1–4 5–7 1–5 7–8 1–8	.000

Mean scores based on a 5-point. Likert’s scale: 1 = not at all. 5 = very much. n.s. = not significant. Number of the respondents groups: *1* farmers; *2* expert–scientist; *3* employees of the national park landscape parks and Public Forests; *4* representatives of local governments; *5* tourists and owners of summer houses; *6* members of pro-ecological and art organizations; *7* teachers working in the researched regions; and *8* college students from those regions; ANOVA [*n* = *230; α* = 0.05; *F*_crit_ = 2.051]

In the case of the future Roztocze–Solska Forest BR, the participants proposed approximately 60 landscape features which should be taken into consideration, while identifying LQOs (Table 1, Column 6). The highest number of points was given to: (1) a multi-stripe field mosaic with lines of numerous balks overgrown with weeds and numerous clusters of trees and shrubs; (2) diverse mosaic of small patches of fields and forests; (3) vast complexes of a high variety of natural forests; (4) historical urban-landscape structures of the former Zamoyski Estate Quarries; and (5) a typical, undulating, upland landscape (Chmielewski and Sowińska 2006, 2008). The highest difference in means occurred in the case of biotic features (difference = 2.07), while a considerable agreement was reached with respect to abiotic and scenic beauty features (Table 3, part A). Similarities of opinion concerning the landscape physiognomy may be explained by landscape esthetic theory, according to which preferences of visual landscape characteristics are considered to be less related to cultural and personal background (Kaplan and Kaplan 1989). Just like in the Polesie BR, the opinions represented by farmers differ the most from the views expressed by other groups.

**Table 3:** Attitudes of Roztocze–Solska Forest Biosphere Reserve respondents toward main landscape features and its threats

Factor	1	2	3	4	5	6	7	8	*F* (7,232)	Significant difference between means			Significance *P*<
										*P* < 0.05	*P* < 0.01	*P* < 0.001	
A. Main characteristic landscape features													
Abiotic features													
Characteristic, undulating, upland landscape	3.13	3.33	3.77	3.97	3.90	3.43	3.37	3.70	1.73	–	–	–	n.s.
Plentiful of springs and clean rivers	3.50	3.67	3.37	3.90	3.57	3.23	3.37	4.07	1.36	–	–	–	n.s.
Biotic features													
Vast complexes of diverse natural forests	4.83	4.27	4.43	3.33	2.77	3.73	3.93	4.07	10.21	3–6	1–8 4–8 2–4 5–6	1–4 1–7 3–5 1–5 2–5 5–7 1–6 3–4 5–8	.000
Cultural													
Multi-stripe field mosaic with lines of numerous balks overgrown with weeds and numerous clusters of trees and shrubs	3.97	4.77	4.70	4.13	4.10	4.97	4.03	3.77	6.64	2–3 3–4 3–5	1–2 3–7 1–3 3–8 2–7	1–6 5–8 2–8 6–7 4–6 6–8 5–6	.000
Historical fields structure of West Roztocze	3.37	4.00	4.40	3.40	3.83	4.10	3.47	3.70	3.09	1–2 3–8 1–6 4–6 2–4 6–7	–	1–3 3–4 3–7	.004
Regional wooden rural architecture	3.63	3.07	3.37	3.23	3.23	2.90	3.60	3.63	1.47	–	–	–	n.s.
Small area rural and provincial colonization	2.87	3.00	2.70	2.87	2.80	2.70	3.13	3.30	0.89	–	–	–	n.s.
Religious sanctuaries, monuments of religious architecture, and roadside and riverside chapels	4.97	3.53	3.63	3.87	3.43	3.43	3.27	3.37	6.10	–	–	1–2 1–6 1–3 1–7 1–4 1–8 1–5	.000
Historical urban-landscape structures of the former Zamoyski Estate Quarries	3.30	3.57	3.60	4.03	4.27	4.40	3.50	3.70	3.06	2–5 3–5 3–6 5–7 6–8	1–4 1–5 2–6 6–7	1–6	.004
Scenic beauty													
Diverse mosaic of small patches of fields and forests	3.90	3.97	4.17	4.10	4.03	3.73	3.67	4.17	0.65	–	–	–	n.s.
Diverse mosaic of forest, peat bog, meadow, and steppe ecosystems	3.37	3.37	3.60	3.10	3.53	4.03	3.20	2.77	2.44	1–6 1–8 3–8 5–8	4–6 6–7	6–8	.020
Vast, open spaces of fields	3.57	2.77	2.73	2.97	2.83	2.80	2.73	2.43	1.99	–	–	–	n.s.
B. Main threats to characteristic landscape features													
Devastation of the natural structure of water bodies and transformation of river valleys	3.87	3.20	3.43	3.07	3.43	3.20	3.33	3.90	1.51	–	–	–	n.s.
The sprawl of habitable buildings and summer cottages over the open space of fields and meadows	3.37	4.03	3.90	3.83	4.03	4.10	4.03	4.17	1.31	–	–	–	n.s.
Vanishing of features typical of rural architecture	3.73	2.77	2.97	3.67	3.23	3.00	3.10	2.87	2.25	1–3 3–4 1–8 4–8	1–2	–	.031
Construction of cell-phone towers and wind-power plants at the most exposed view-points	2.70	3.43	3.70	3.33	2.80	3.43	2.77	3.07	2.22	1–2 2–7 1–3 3–7 1–6	–	–	.033

Mean scores based on a 5-point. Likert scale: 1 = not at all. 5 = very much. n.s. = not significant. Number of the respondents groups: *1* farmers; *2* expert–scientist; *3* employees of the national park, landscape parks and Public Forests; *4* representatives of local governments; *5* tourists and owners of summer houses; *6* members of pro-ecological and art organizations; *7* teachers working in the researched regions; and *8* college students from those regions; ANOVA [*n* = 240; *α* = 0.05; *F*_crit_ = 2.049]

With regard to the second question of the questionnaire, representatives of the local community in the West Polesie BR attributed the highest importance to three threats: (1) the disappearance of wetlands and drying of peatbogs and bogs as well as regulation of river beds (27.27 %); (2) the location of tourist housing on lakesides (25.68 %); and (3) the disappearance of open-space peatbogs and meadows (22.22 %) (Table 4, Column 3). It is not surprising that the significance of those threats was so highly emphasized by the respondents. Hydrogenic landscapes constitute a distinguishing mark of the Polesie and are a major factor attracting tourists and artists. They determine the unique natural values of the region. Furthermore, preferences for landscape with high ecological values are associated with attitudes that are protective of this natural resource (Williams and Cary 2002). Surprisingly, in the opinion of all the groups, threats to other landscape components are almost insignificant (<1 % of the total number of points). The differences of opinions between groups of respondents were statistically significant (Table 2, part B). For example, members of pro-ecological and art organizations gave a considerably lower rating to the disappearance of wetlands, drying of peatbogs and bogs, and regulation of river beds in relation to other groups.

**Table 4:** Comparative assessment of the social opinion on threats to the landscape quality of the West Polesie Biosphere Reserve and the future Roztocze–Solska Forest Biosphere Reserve

1. Landscape components	2. Social opinion on the main threats to the quality of landscape components	West Polesie		Roztocze–Solska Forest	
		3. % of total number of points	4. Threat Rank Index	5. % of total number of points	6. Threat Rank Index
Aquatic and meadow ecosystems	Disappearance of wetlands, drying of peatbogs and bogs, as well as regulation of river beds	27.27	7	–	5
	Location of tourist housing on lakesides	25.68		–	
	Devastation of the natural structure of water bodies and transformation of river valleys	–		21.51	
	Disappearance of open-space peatbogs and meadows, taken over by forests and construction	22.22		–	
	Improper management of new water bodies	0.03		–	
	Meadow burning	0.03		0.25	
	Overgrowth of meadows as a result of natural succession	–		0.05	
		Σ 75.24		Σ 21.81	
Forest ecosystems	Forest cutting	–	4	1.18	4
	Illegal wastes dumping in forests	0.25		1.45	
		Σ 025		Σ 2.63	
Field ecosystems	Elimination of balks	–	3	0.52	2
	Cutting down of mid-field tree clusters	–		0.27	
	Vanishing of rural dirt roads and elimination of balks	0.04		–	
	Wastelands	0.04		–	
		Σ 0.08		Σ 0.79	
Flora and fauna	Disappearance of rare and protected species	0.02	1	0.02	1
	Devastation of flora as a result of tourist pressure	0.02		0.02	
		Σ 0.05		Σ 0.04	
Land use	The sprawl of habitable buildings and summer cottages over the open space of fields and meadows	23.27	6	25.90	7
	Construction of roads constituting ecological barriers	–		0.91	
	Location of housing near protected areas	0.15		–	
	Power lines	–		0.14	
	Industry	0.03		–	
		Σ 23.51		Σ 26.95	
Cultural heritage	Vanishing of features typical of rural architecture	–	5	20.58	6
	Contamination of environment components	0.40		2.58	
	Disharmonious dwelling and industrial buildings	–		0.41	
	Improper management of the environment of sites of historical value	0.43		–	
	Devastation of monuments of cultural heritage	–		0.22	
	Omnipresent advertising billboards	0.03		–	
		Σ 0.86		Σ 23.79	
Other	Construction of cell-phone towers and wind-power plants at the most exposed view-points	0.03	2	22.07	3
	Uncontrolled tourism	–		0.93	
	Noise	0.04		0.80	
	Non-effective nature conservation management	–		0.19	
		Σ 0.07		Σ 23.99	

Representatives of the local community of the future Roztocze–Solska Forest BR, on the other hand, considered land use transformations as the greatest danger, particularly the sprawl of habitable buildings and summer cottages over the open space of fields and meadows (25.90 %). A very high number of points (more than 20 %) were also attributed to such processes as the construction of cell-phone towers and wind-power plants at the most exposed view-points, devastation of the natural structure of water bodies as well as the transformation of river valleys, and vanishing of features typical for rural architecture (Table 4, Column 5). Just like in the other study area, those threats are related to the characteristic elements of the analyzed region: traditional rural landscape of Roztocze and small river valleys of Solska Forest. The opinions on two main threats to the characteristic landscape features were similar between groups. However, statistically significant differences between means of the two others were reported (Table 3, part B). Analogously to the first question, the most different opinions were from farmers and, in the case of Polesie BR, also members of pro-ecological and art organizations. The farmers gave the lowest rating to the location of tourist housing on lakesides and the expansion of buildings and summer cottages over the open space of fields and meadows.

### Systematization of Landscape Features and Its Threats

Twenty-three landscape components have been defined within the four major categories: abiotic, biotic, cultural, and physiognomic. Additionally, the fifth group of characteristic elements which did not fit into the four main categories has been defined. These elements are related to land use activity and include nature conservation, agriculture, and tourism. Thus, the final list consists of 26 components (Table 1, Column 2). The authors are aware that the list does not contain the full spectrum of landscape components. It only includes the attributes which in public opinion are crucial for the quality of landscape in the areas investigated and help determine their physiognomic identity.

The analysis of the second part of the questionnaire permitted us to define six main components of the landscapes investigated which in public opinion are considered to be susceptible to transformation and degradation. They are as follows: aquatic–meadow ecosystems, forest ecosystems, field ecosystems, flora and fauna, settlements, and cultural heritage (Table 2, Column 1).

### Comparative Assessment of the Public Opinion on Landscape Quality and Its Threats

**Fig. 5:**
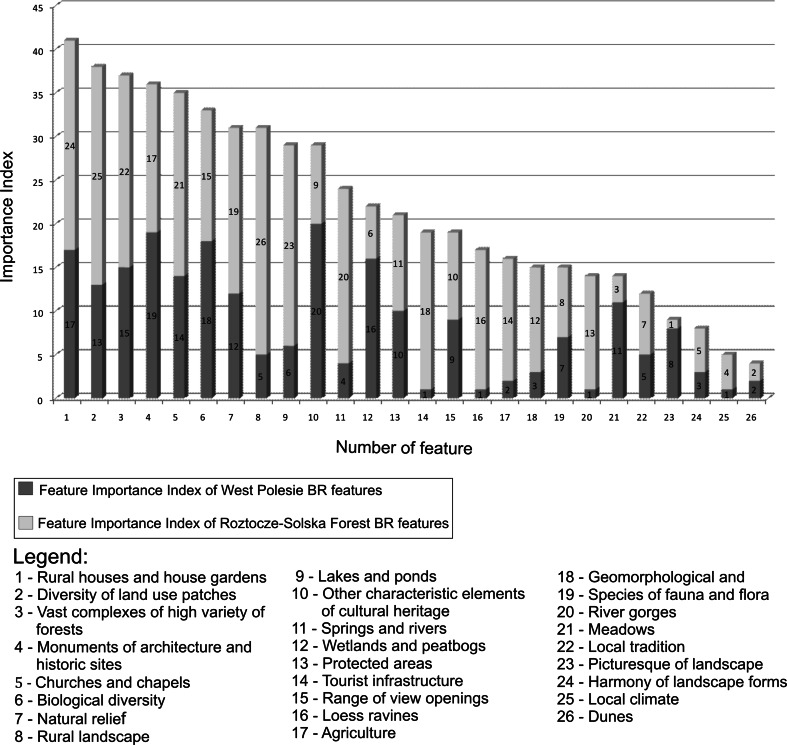
Feature Importance Index (FII) ascribed to each landscape feature by representatives of the local communities of the West Polesie BR and the future Roztocze–Solska Forest BR, according to the gradation of the Total Importance Index

The numerical values of the TFII revealed that representatives of local communities of both reserves consider the following features to be most important for quality of landscape:

- rural houses and house gardens (regional forms of architecture surrounded by traditional gardens);
- diversity of land use patches (a mosaic of different kinds of ecosystems);
- vast complexes of diverse natural forests;
- monuments of architecture and historical sites;
- churches and chapels (as symbols of the multi-cultural history of both regions);
- biological diversity (Fig. 5).

The results show that the FIIs of eight abiotic components of the landscape differ significantly between both regions (Table 1, Column 5 and 8). Sand dunes constitute an exception, since they are considered to have minor significance in the character of landscape of both areas investigated. The social underestimation of this abiotic landscape component may be due to the fact that dunes occurring in both Polesie and Roztocze–Solska Forest are almost entirely wooded and therefore may be identified with forest ecosystems instead of an abiotic element of the natural environment. The FIIs ascribed to elements of landscape physiognomy were also remarkably dissimilar. For example, participants from the Roztocze–Solska Forest BR found view openings as crucial for the quality of landscape and therefore worthy of preservation. Respondents from the other region expressed no opinion on this issue.

More obvious similarities are observed in the case of the biotic component group. Two out of five features from this category have a similar FII, specifically species of fauna and flora (7/8) and biological diversity (18/15). In spite of remarkably different character of landscapes in the areas investigated, very analogous expectations exist with respect to most features of cultural heritage, such as churches and chapels, monuments of architecture and historical sites, or local tradition. One exception is rural landscape, in which case the index differentiation is the highest among all 26 components, and reaches 21 points. Within the group of other typical elements, the protected areas and tourist infrastructure have similar FIIs (10/11 and 9/10, respectively), while agriculture FIIs differ considerably (2/14).

**Fig. 6:**
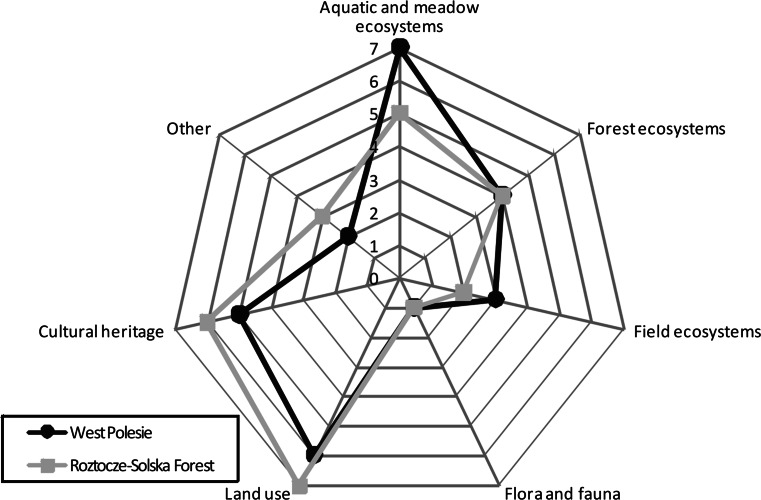
Threat Rank Index (ThRI) values given to each landscape component by representatives of the local communities of the West Polesie BR and the future Roztocze–Solska Forest BR

The comparative assessment of public opinion regarding major landscape threats revealed that despite the variety of classes and types of natural landscapes, the environment of both BRs under study is in most cases subject to the same development forces. In the opinion of representatives from both regions, the highest ThRI values concern transformation of aquatic–meadow ecosystems, land use changes, and disappearance of typical elements of cultural heritage, particularly traditional forms of rural architecture (Fig. 6, values 5, 6, or 7). The lowest Threat Rank Index values concern endangerment of flora and fauna, as well as field ecosystems (values 1, 2, or 3). Comparison of these results with Total Feature Importance Index values reveals certain similarities. Elements such as cultural heritage and aquatic–meadow ecosystems, considered particularly vulnerable by the participants, were also highly rated as important to the character of the landscape of both regions. According to public opinion, the feature referred to as fauna and flora and its endangerment received a very low rating.

## Discussion

### The Importance of Sociological Research

The results, frequently unexpected and surprising for the authors, confirm the main objective of the public participation approach. Thus, we can extend the scientific expertise by adding local experiences, opinions, knowledge about the landmarks, perceived threats to characteristic landscape features, and processes which have impact on the physiognomy of a given area (Arler 2000; Majchrowska 2011; Sevenant and Antrop 2010). Our study once more confirms that different social and occupational groups of people look at landscapes in a different way, attaching importance to different landscape features and finding different threats to its quality (Strumse 1996; Eisler et al. 2003; Tveit 2009). On the one hand, the tourists, as people who only occasionally visit a given area, are the first to notice even small landscape physiognomy changes and first signs of land use transformations (Herzog et al. 2000). Such changes may occur imperceptibly to local inhabitants and authorities. This was evidenced by the research conducted. It was tourists who noticed the highest number of threats to landscape quality, in comparison with other groups. On the other hand, only the inhabitants and people originating from a particular region have a detailed knowledge of the quality of the environment. In many cases, this knowledge cannot be achieved by people who are not emotionally connected with the land (Rogge et al. 2007).

The inhabitants can “open our eyes to qualities we have not been aware of before and they can make us rethink our own experiences and preferences” (Arler 2000). Moreover, the approach of dividing the “general public” into farmers and other groups living in the countryside proved justified, because the farmers expressed different opinions from other Polesie and Roztocze residents. This phenomenon was also reported in a number of recent studies (Natori and Chenoweth 2008; Nijnik and Mather 2008; Rogge et al. 2007). A possible explanation is that the farmers represent a different approach to landscape from other social groups. Agricultural land is the place of their work and a source of their livelihood. Their preferences may express their expectations of what the land could offer, in the terms of its benefits (Purcell et al. 1994). Thus, ‘non-profitable’ features such as inaccessible, virgin lakes, or vast complexes of diverse natural forests are rated lower by this social group. The farmers also gave a very low rating to the landscape threats affecting the scenic beauty, such as the location of tourist housing on lakesides and the expansion of buildings and summer cottages over the open space of fields and meadows. The reason may be that farmers consider the landscape physiognomy values of their region as something mundane and ordinary. Other groups of respondents, due to their professional background and technical knowledge, notice the negative visual impact of building expansion and are aware of the threats to natural resources which follow that transformation. Moreover, the threat of land development is perceived by the farmers from the economic point of view, since they are not only responsible for selling the land to tourists, but also for building summer cottages in the fields and meadows. The social cross section of people interviewed in this study seems to be adequate for future studies and aimed at the identification of LQOs at the regional level. The groups interviewed include all types of respondents, so-called stakeholders, affecting public decisions (Nijnik and Mather 2008; Sevenant and Antrop 2010; Van Asselt et al. 2001).

The comparative assessment of the public opinion regarding quality of landscape revealed certain distinct differences as well as universal likenesses to other countries. The feature described as diversity of landscape (as a result of a complex mosaic of different types of ecosystems and land use forms) was given a very high rank by the interviewees from both investigated areas and was generally highly appreciated by the representatives of diverse target groups from different countries (Nijnik et al. 2008; Rogge et al. 2007; Sayadi et al. 2009). Surprisingly, the Polish results also suggested that features relating to cultural heritage are closely associated with the unique character of both study areas, whereas notably less importance was given to the characteristic features of abiotic components. These findings revealed certain distinct differences from another eastern European countries, namely Ukraine, where inanimate components are considered to be valuable elements of the countryside landscape (Nijnik et al. 2008). This difference may be attributed to the fact that predominant number of participants live in or originate from the investigated areas. In their understanding, the landmark of their home town is a monument of architecture of national importance, and not an abiotic component of landscape, such as geomorphological forms or local climate. Polish citizens from both analyzed regions are generally not aware that inanimate elements of landscape can have a unique value and therefore are worthy of including them in the process of LQOs identification. Moreover, in public opinion, many regions are particularly associated with their cultural symbols, such as historical churches, unique castle complexes, or traditional, vernacular homes, and villages (Sayadi et al. 2009). In view of the conducted research, this tendency is particularly noticeable in the case of the Roztocze–Solska Forest region, where the historical building of the former Zamoyski Estate, as well as religious sanctuaries and riverside little chapels, is the most popular destination point of tours.

The importance of public opinion was also emphasized, while discussing the results of the second part of the questionnaire. The outcome of a telephone survey conducted with 213 managers of BRs, carried out by Mehring and Stoll-Kleemen (2008), proved that the greatest threats to biosphere reserves are illegal activities, as well as overexploitation, and land use changes (modification of the natural environment into a developed area). In wooded BRs in high-income countries, the threats also included climate changes, invasive species, and tourism. Most opinions from both BRs were also related to land use changes (i.e., the transformation of agricultural landscape; the disappearance of open spaces, water spots, and flood zones; and the construction of rest and recreation facilities) and illegal activities (uncontrolled development of summer housing; meadow burning; and waste dumping in forests). It is noteworthy that the problems of climate change and invasive species were not mentioned even by the representatives of the expert–scientist group or employees of the national park, landscape parks, and Public Forests. The reason may be that the current transformation of land is very rapid and has a direct impact on human well-being, whereas the consequences of climate change and invasive species are spread over time. It is worth emphasizing that the respondents also pointed to threats resulting from improper management of the reserves, mainly in relation to water ecosystems and historical sites. The threats resulting from inconsiderate decisions of local governments were also mentioned, such as regulation of river beds, public forest cutting, and construction of cell-phone towers and wind-power plants at the most exposed view-points.

To sum up, the results from our study once again strongly justify the need to incorporate the opinion of local communities in any type of work related to landscape issues, particularly in LQOs identification. The participatory approach is deemed suitable to enable research to fully understand values of a given area and to define and analyze processes occurring in natural and cultural environments.

### Landscape Preferences Versus Landscape Type

Given the demands of landscape policy, as formulated in the European Landscape Convention, a conceptual base is needed to characterize the landscape according to public preferences. The results of the present study confirm the difficulty of developing such a framework since landscape preferences related to some categories differ not only among participant groups but also among landscape types. Hence, in the process of recognition of landscape perception and evaluation, the ties between different preferences in varying landscape types should be fully analyzed (Sevenant and Antrop 2010). The problem of the nature of inter-relation between public preferences and landscape types was noticed by many authors. According to Tveit et al. (2006), there is a positive correlation between these two factors, because some concepts enforce each other and others cancel each other out. In turn, results from the studies by Coeterier (1996) and Purcell (1992) indicated that one landscape component which may be typical for a given landscape type may not play a predominant role in comprehensive landscape validation. Analogously, a study conducted by Sevenant and Antrop (2010) showed that the recorded ratings varied between different landscape vistas. Findings from the study conducted by Byoung and Brown (1992) suggested that, regardless of cultural differences, landscape style typical of a given geographic area is the factor with the greatest influence on landscape preference. In our study, the comparative assessment revealed that similar social expectations of landscape quality exist with regard to cultural heritage, protected areas, tourist infrastructure, and some biotic components. These preferences differ significantly depending on the class and type of natural landscapes (where the abiotic components are dominating criteria in the classification process). The assessment also showed that the fundamental factor in the rating of abiotic components is the differentiation of land relief (Roztocze region) and the abundance of resources as well as diversity of surface water (both analyzed regions). This finding may also explain why interviewees from the Roztocze–Solska Forest region come up with more ideas of landscape features and threats and described them in more detail than the participants from the other reserve. This observation does not simply reflect the people’s knowledge and ecological awareness, but results from the fact that the future Roztocze–Solska Forest BR is much more diverse in terms of land use and physiognomy than the West Polesie BR. Consequently, it may be easier to specify numerous features worthy of conservation, related to diverse landscape components.

The results of the Polish study are only partially parallel to the findings of Coeterier (1996), who defined a limited set of attributes to explain landscape perception, regardless of the landscape type. These include the landscape unity, function, maintenance, naturalness, spaciousness, development in time, soil, water, and sensory qualities. Among them, only features related to the function and maintenance were similarly rated by the representatives of both investigated regions of Poland. Moreover, the results from our study suggested that the diversity and cultural heritage values should also be included in the Coeterier’s list of attributes. Diversity, construed as complexity and variation, was also indicated as one of the main attributes by Sevenant and Antrop (2009) and the historical character by Galindo Galindo and Corraliza Rodriguez (2000).

### Future Outlook

The knowledge of social expectations regarding landscape quality is crucial for effective landscape management on the sub-regional and local level. Successful protection of landscapes is possible only under conditions of wide social acceptance. In Poland, the overall spatial structure of land use depends on local government decisions with regard to spatial planning, while the spatial arrangement and the physiognomy of an individual farm depend on an individual resident. On the one hand, it is necessary to know public expectations regarding the desirable landscape quality and on the other hand, landscape education is needed. Poland has significant achievements in the field of ecological education, but the educational framework, methods, and techniques of landscape education are only in the initial phase (Chmielewski 2012). Results of the study may contribute to the development of such methods.

It is crucial for the development of LQO identification methodology that features which obtained the highest TII were highly rated by the representatives of both investigated regions. It can be concluded, that in spite of different landscape types, it is possible to distinguish a set of typical features decisive for the character of landscape and common for both areas discussed. Those features should be primarily considered in the process of LQO identification. Such generalization makes it possible to shift from regional to national scale studies and to identify LQOs by using homogenous criteria for the entire territory of Poland. In order to verify and refine these criteria, it is advisable to perform a similar analysis on more sample areas belonging to other classes and types of natural landscape and other cultural regions, and, on the basis of collected data develop a list of most important landscape features which in public opinion require protection. However, upgrading a set of typical Polish landscape features to European scale needs to be conducted carefully. Landscapes of each country differ in unique land relief forms, typical land cover, ecological resources, historical land use, and cultural heritage values. Some features characteristic for the landscape of a given country may be typical for another area as well, but they can also be absent in that region. Secondly, preferences of the same social or occupational group may vary significantly from one country to another due to the cultural, social, and economic conditions. To make a generalization and distinguish features which in public opinion require protection in the entire European territory, an analogous sociological research should be done for each country. The result would show if it is possible to use homogenous criteria for LQO identification for many countries. Carrying out such comparative studies would be very interesting also from a social point of view.

The conducted research is valuable beyond European borders as well, especially for the biosphere reserves on other continents, not covered by the ELC. Results of the study revealed that in spite of the application of the LQO concept, comparative assessment gives insight into the way in which people perceive different types of landscape and assess threats to its characteristic features. Such recognition helps to prioritize actions for a long-term management of a biosphere reserve. Public participation is inherent to the concept of a BR, since each reserve represents the interdependence of society and nature in a socio-ecological system (Parrot et al. 2012; Welp 2000). As demonstrated by the results of a global survey conducted by Stoll-Kleeman and Welp (2008), reserve managers consider community participation as a critical component in successful conservation and management. The management style based on communication and cooperation between representatives of different sectors and inhabitants, called “management as mutual learning,” seems to be most appropriate for biosphere reserves. A review conducted by Hirschnitz-Garbers and Stoll-Kleemann (2011) shows that local people’s participation ensures the continued survival of many protected areas by leading to better decision making, as local people are likely to feel responsible for conservation rather than resistance to it. Besides, the recognition of perceptions and attitudes of local residents toward landscape are vital for successful management because protected areas cannot coexist with communities that are hostile to them. Furthermore, since two BRs were used as test polygons for a new method application, our study contributes to the strengthening of BRs’ role as a worldwide network of areas of special importance for scientific and ecological research, labeled by Nguyen et al. (2011) as ‘Learning Laboratories for Sustainable Development’.

## Conclusions

Considering the research objectives established by the authors at the beginning of the study, the comparative assessment led to the following conclusions:

1. In spite of a significantly different character of landscape in the regions investigated, similar social expectations of landscape quality exist with regard to features related to cultural heritage, protected areas, tourist infrastructure, and partly to the land use structure;
2. public expectations differ significantly depending on the class and type of natural landscapes (abiotic components);
3. representatives of both local communities consider the following features as most important to the preservation of the local landscape identity: (a) rural houses and house gardens; (b) diversity of land use patches and their characteristic spatial arrangement; (c) vast complexes of diverse natural forests; (d) monuments of architecture and historical sites; and (e) churches and chapels;
4. in public opinion, landscapes of both investigated regions are in most cases subjected to the same negative forces of transformation and development pressures. Despite a totally different character of landscape in both investigated regions, the greatest threats to landscape quality are similar and include: (a) land use changes (especially urban sprawl); (b) vanishing of typical elements of cultural heritage; and (c) transformation of aquatic, peat, and meadow ecosystems;
5. the knowledge of public opinion concerning the need of nature protection and achieving the desirable landscape quality is essential for long-term management of both regions.
